# TNF-α blockade for carbamazepine-induced drug reaction with eosinophilia and systemic symptoms

**DOI:** 10.3389/fimmu.2026.1872304

**Published:** 2026-08-03

**Authors:** Min Zou, Li Xue, Dengmei Xia, Kun Zhan, Jishu Li, Xun Feng, Wei Yan, Wei Li

**Affiliations:** 1 Department of Dermatology and Venereology, West China Hospital, Sichuan University, Chengdu, China; 2 Department of Dermatology & Rare Diseases Center, West China Hospital, Sichuan University, Chengdu, China; 3 Department of Dermatology, West China Second University Hospital, Sichuan University, Chengdu, China

**Keywords:** carbamazepine, dress, drug-induced hypersensitivity syndrome, etanercept, severe cutaneous adverse reactions, TNF-α inhibitor

## Abstract

**Background:**

Drug reaction with eosinophilia and systemic symptoms (DRESS) is a potentially fatal adverse drug reaction featuring widespread eruption, fever, and multi-organ involvement. Carbamazepine is a leading causative agent in Asian populations. While systemic corticosteroids remain first-line therapy, a subset of patients exhibits corticosteroid-refractory or corticosteroid-dependent disease. Elevated tumor necrosis factor-alpha (TNF-α) levels during the acute phase provide a rationale for TNF-α blockade as an adjunctive treatment, yet clinical experience with TNF-α blockade in DRESS remains limited.

**Objective:**

To explore the potential effectiveness and safety of TNF-α inhibitors as an adjunctive treatment for DRESS.

**Methods:**

We retrospectively reviewed three consecutive inpatients diagnosed with carbamazepine-induced DRESS (RegiSCAR scores 5–9) at our dermatology department between September 2025 and March 2026. All received subcutaneous etanercept biosimilar, with individualized dosing. A systematic literature search was also performed to summarize previously published cases of DRESS treated with TNF-α inhibitors.

**Results:**

In our cohort, the three patients represented distinct phenotypes, and all had responded inadequately or transiently to corticosteroids alone. The addition of etanercept biosimilar was associated with clinical stabilization, progressive cutaneous improvement, and improvement in hepatic enzymes. Hospital stays ranged from 16 to 20 days with no etanercept-related adverse events. The literature review identified 8 previously reported DRESS cases treated with TNF-α inhibitors, the majority of which demonstrated a therapeutic response.

**Conclusion:**

Combined with previously published cases and our single-center experience, these findings support the potential benefit and an acceptable short-term safety profile of TNF-α inhibitors in this limited DRESS cohort.

## 1 Introduction

Drug reaction with eosinophilia and systemic symptoms (DRESS) syndrome, also referred to as drug-induced hypersensitivity syndrome (DIHS), is a rare but potentially life-threatening severe cutaneous adverse reaction (SCAR) characterized by widespread morbilliform eruption, fever, lymphadenopathy, hematologic abnormalities (particularly eosinophilia and atypical lymphocytosis), and multi-organ involvement (1). Its precise population incidence remains uncertain and varies according to the study population, causative drugs, and case definitions. Reported estimates include a prevalence of 2.18 per 100, 000 patients in a large US electronic health record cohort from 1980 to 2016, and mortality has generally been estimated at 5%–10% (2, 3). Aromatic antiepileptic drugs—most notably carbamazepine, phenytoin, and phenobarbital—are among the most frequently implicated causative agents, particularly in Asian populations (2, 4).

DRESS is characterized by a complex immunopathogenesis that is not yet fully understood, involving the activation of CD8^+^ and CD4^+^ T cells and the production of proinflammatory cytokines, including TNF-α, IFN-γ, IL-4, IL-5, and IL-13, which drive eosinophilia (4). Genetic susceptibility also contributes, as individuals carrying high-risk HLA alleles are more prone to developing drug-specific hypersensitivity reactions (2, 4, 5).

The cornerstone of DRESS management remains prompt discontinuation of the offending drug and initiation of systemic corticosteroids, and intravenous immunoglobulin (IVIG) is frequently added in severe or refractory cases (6, 7). Nevertheless, a substantial subset of patients exhibits an inadequate or transient response to corticosteroids alone. TNF-α inhibitors, such as infliximab and etanercept, have emerged as a potential salvage option in corticosteroid-refractory (persistent or progressive cutaneous lesions and/or organ involvement despite systemic corticosteroid-based therapy) and corticosteroid-dependent (relapse during corticosteroid tapering or inability to taper corticosteroids without recurrence) DRESS (8), while evidence is restricted to limited case reports (9, 10).

Here we report three Chinese patients with carbamazepine-induced DRESS who received a TNF-α inhibitor (recombinant human TNFR2-Fc fusion protein, etanercept biosimilar) as part of their inpatient management at our dermatology department. Combined with previously published experience, these cases illustrate the clinical rationale and therapeutic potential of TNF-α blockade across the DRESS clinical spectrum.

## 2 Materials and methods

### 2.1 Case series

This is a single-center retrospective case series conducted at the Department of Dermatology, West China Hospital, Sichuan University, China. We identified consecutive inpatients who fulfilled the following criteria between September 2025 and March 2026: (1) diagnosis of DRESS based on the European Registry of Severe Cutaneous Adverse Reactions (RegiSCAR) scoring system (11), with a score ≥4 (probable or definite); (2) treatment with an etanercept biosimilar (recombinant human TNFR2-Fc fusion protein) as part of the inpatient regimen; and (3) complete clinical documentation available for review. In PICO terms, this study evaluated adjunctive TNF-α blockade in hospitalized patients with carbamazepine-induced DRESS, with clinical and laboratory improvement, relapse, and treatment-related adverse events as outcomes, and no formal comparator was included because of the retrospective case-series design. Three patients satisfied all criteria and constitute the study cohort. The study was approved by the institutional review board (No. 2025-1851), and written informed consent was obtained from all patients.

DRESS diagnosis and severity classification followed the validated RegiSCAR criteria, which assign scores across these domains: fever (≥38.5 °C), lymphadenopathy, eosinophilia, atypical lymphocytosis, skin rash extent and morphology, organ involvement, and exclusion of alternative diagnoses, etc. (11) A total score of 2–3 is classified as possible, 4–5 as probable, and ≥6 as definite DRESS.

Etanercept biosimilar dosing was individualized at the discretion of the treating team, guided by disease severity, extent of organ involvement, response to prior corticosteroids, and body weight. A weekly 50 mg loading approach was used in patients with more extensive or fulminant disease (Cases 1 and 2), whereas a 25 mg twice-weekly schedule was used for less extensive disease (Case 3) and for maintenance.

Clinical, laboratory, and pharmacological data were extracted from electronic medical records. Extracted variables included demographics, underlying conditions, implicated drug and latency period, RegiSCAR score, laboratory values (white blood cell count, eosinophil count and differential, alanine aminotransferase (ALT), aspartate aminotransferase (AST), gamma-glutamyl transferase (γ-GT), lactate dehydrogenase (LD), serum creatinine, T-lymphocyte subset counts, total IgE, HLA-B*15:02 genotyping, imaging findings, corticosteroid doses, etanercept biosimilar regimen, and clinical outcomes, etc.

### 2.2 Literature review

With the objectives of comparative case aggregation and mechanistic insight, we also conducted a literature search in PubMed and Web of Science from database inception to 1 July 2026 to identify published cases of DRESS/DIHS treated with TNF-α inhibitors. And the detailed search strategy is recorded in **Supplementary Table 1**. Reference lists of eligible articles were manually screened. Reports were included if TNF-α blockade was used therapeutically for DRESS/DIHS. Cases not meeting RegiSCAR criteria for possible, probable, or definite DRESS/DIHS (for patients whose exact RegiSCAR scores were not reported by the original authors, eligibility was assessed by two independent reviewers to determine whether the RegiSCAR diagnostic criteria were met), non-DRESS SCAR cases, and articles without accessible full text were excluded.

## 3 Results

### 3.1 Case series

#### 3.1.1 Case 1

A 38-year-old male with a 6-month history of epilepsy presented with a generalized eruption and fever after approximately 10 days of carbamazepine exposure. The rash progressed rapidly, accompanied by fever (maximum 39.5 °C), myalgia, and productive cough. Despite initial treatment at an outside facility with antibiotics (vancomycin), hepatoprotective agents, and antihistamines, the eruption worsened. Subsequent investigations at the referring facility revealed a peak ALT of 1, 669 U/L, and chest CT showing bilateral pulmonary nodules. The patient developed acute kidney injury (AKI), pericardial effusion, and bilateral pleural effusions within one week. At another referral center, intravenous methylprednisolone sodium succinate (80 mg/day) combined with IVIG (30 g/day × 3 days) improved body temperature but failed to halt skin lesion progression, prompting transfer to our department.

**Figure 1:**
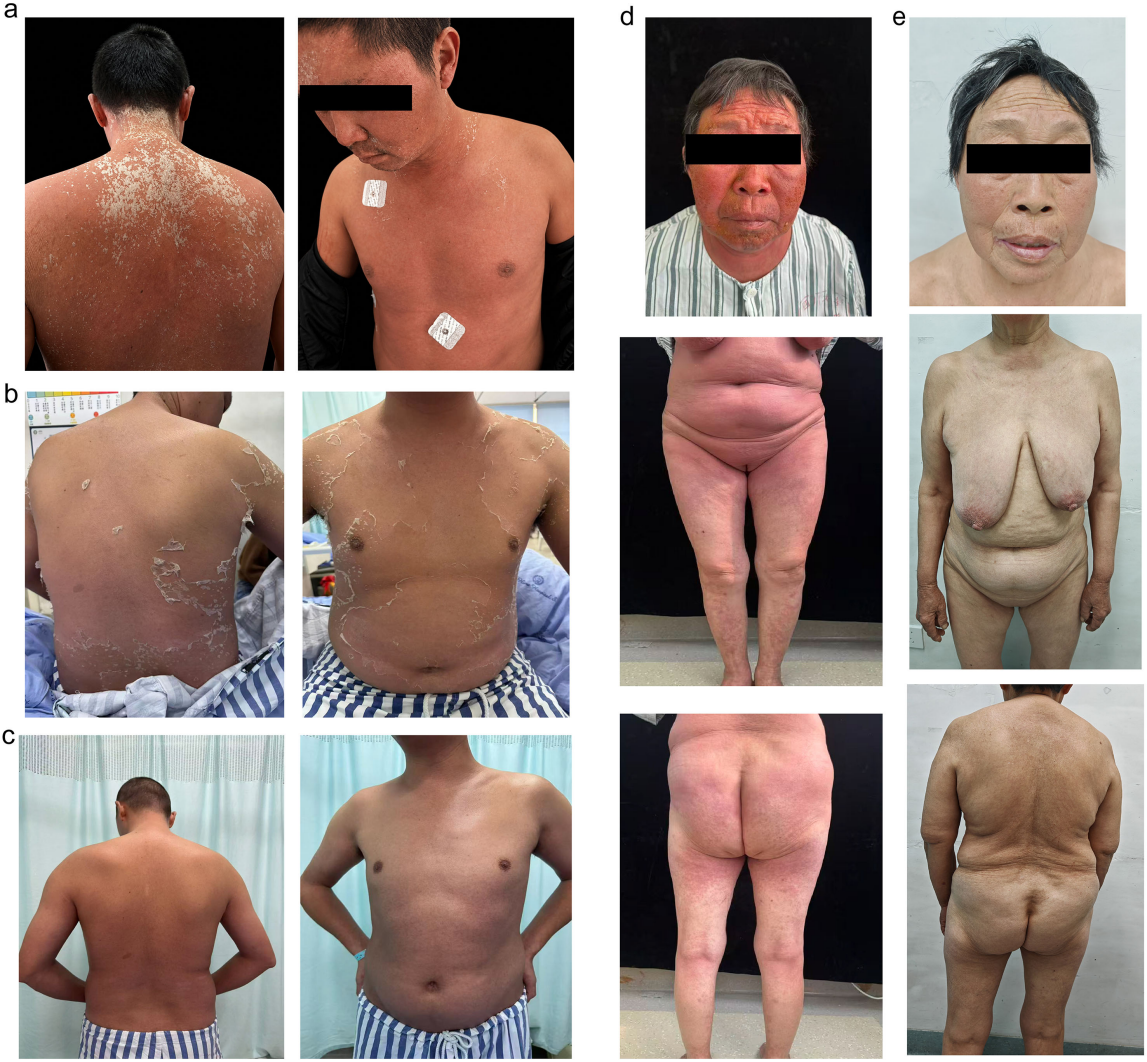
Clinical images of patients. **(a)** Erythema with focal superficial epidermal detachment of Case 1 on admission; **(b)** 3 days after the initial administration of the TNF-α inhibitor, significant resolution of cutaneous lesions was observed, characterized by fading erythema and emerging desquamation (Case 1); **(c)** Complete resolution of cutaneous lesions of Case 1 at discharge; **(d)** Erythema and scattered papules on the face, trunk, and extremities of Case 3 on admission; **(e)** Complete resolution of cutaneous lesions of Case 3 at discharge.

On admission, physical examination revealed confluent erythematous macules and papules involving 50% body surface area (BSA) with focal superficial epidermal detachment, prominent bilateral periorbital erythema, facial scaling, and a right heel ulcer (**Figure 1**). Warmth was noted over the more intensely inflamed, confluent erythematous areas. Additionally, the patient also reported moderate myalgia. Laboratory investigations demonstrated leukocytosis, marked eosinophilia, reactive lymphocytes 15.0% (reference: <2%), significantly elevated CD4^+^ and CD8^+^ T cells, ALT, AST, γ-GT, serum creatinine, etc. (**Table 1**) HLA-B*15:02 genotyping was negative. The RegiSCAR score was 9 (definite DRESS, **Supplementary Table 2**), and carbamazepine was identified as the causative agent.

**Table 1:** Laboratory results and clinical characteristics of patients in our institution.

Variable	Patient 1	Patient 2	Patient 3
Sex/Age (years)	Male/38	Male/62	Female/67
Previous illness	Epilepsy	Trigeminal neuralgia	Trigeminal neuralgia
Latency period	10 days	1 month	3 weeks
HLA-B*15:02	Negative	Negative	Negative
RegiSCAR score	9 (definite)	6 (definite)	5 (probable)
BSA involved (%)	50%	60%	55%
WBC on admission/at discharge (×10^9^/L, Ref: 3.5-9.5)	23.86/6.60	20.30/9.35	32.59/10.14
AEC on admission/at discharge (×10^9^/L, Ref: 0.02-0.52)	12.53/0.12	<0.001/0.11	2.28/0.51
LDH (U/L, Ref: 120-250)	872/194	852/225	290/162
γ-GT (U/L)	882/334 (Ref: 10-60)	41/50 (Ref: 10-60)	279/38 (Ref: 7-45)
ALT on admission/at discharge (U/L)	875/37 (Ref: 9-50)	71/44 (Ref: 9-50)	147/65 (Ref: 7-40)
AST on admission/at discharge (U/L)	164/19 (Ref: 15-40)	20/22 (Ref: 15-40)	73/26 (Ref: 13-35)
Creatinine on admission/at discharge (μmol/L)	123/85 (Ref: 68-108)	557/99 (Ref: 68-108)	86/62 (Ref: 49-88)
eGFR on admission/at discharge (mL/min/1.73m (2))	64/99.7	8.7/70.5	60.4/89.7
Total IgE on admission/at discharge (IU/mL, Ref: <358)	46/72	497/212	1, 103/328
CD4^+^ T cells on admission/at discharge (cells/μL)	3, 955/858 (Ref: 465-1680)	1205/399 (Ref: 338-1125)	6, 515/1617 (Ref: 411-1635)
CD8^+^ T cells on admission/at discharge (cells/μL)	1884/943 (Ref: 284-1221)	503/205 (Ref: 184-1126)	4056/756 (Ref: 180-892)
B cells (cells/μL)	475/140 (Ref: 86-618)	504/162 (Ref: 63-500)	624/337 (Ref: 50-442)
NK cells (cells/μL)	77/304 (Ref: 103-701)	576/134 (Ref: 133-1228)	360/455 (Ref: 97-871)
Prior corticosteroid dose	MP 80 mg/day + IVIG (outside)	MP 40 mg/day (multiple courses)	MP 40 mg/day × 2 days
Max in-hospital CS dose	MP 60 mg/day	MP 60 mg/day	MP 60 mg/day
Etanercept biosimilar regimen	50 mg weekly × 2 + 25 mg twice weekly × 4 maintenance	50 mg weekly × 1 + 25 mg twice weekly × 4 maintenance	25 mg twice weekly × 2
Hospital length of stay (day)	20	17	16

AEC, absolute eosinophil count; ALT, alanine aminotransferase; AST, aspartate aminotransferase; BSA, body surface area; CS, corticosteroid; eGFR, estimated glomerular filtration rate; IVIG, intravenous immunoglobulin; LDH, lactate dehydrogenase; MP, methylprednisolone sodium succinate; RegiSCAR, European Registry of Severe Cutaneous Adverse Reactions; WBC, white blood cell count; γ-GT, gamma-glutamyl transferase.

Carbamazepine was discontinued immediately upon admission. Methylprednisolone sodium succinate (60 mg/day intravenously) was continued with a planned taper. Although the fever resolved within 3 days, cutaneous lesions showed minimal improvement. The etanercept biosimilar was therefore added at 50 mg subcutaneously once weekly (two doses administered during hospitalization), alongside hepatoprotective therapy, topical corticosteroids, and anti-infective support. Significant resolution of cutaneous lesions, characterized by fading erythema and emerging desquamation, along with gradual recovery of hepatic and renal function, was observed within 3 days, a period that coincided with the initiation of TNF-α blockade in the setting of concurrent systemic corticosteroids and supportive care (**Figure 1**). The patient was discharged on hospital day 20, with significant improvement in blood test indicators and near-complete cutaneous lesion clearance. Therapy was maintained on a 4-week regimen of TNF-α blockade therapy (25 mg twice weekly), along with prednisone 30 mg daily with a gradual taper. No relapse was observed within 6 months.

#### 3.1.2 Case 2

The patient, a 62-year-old male with a history of trigeminal neuralgia, had been receiving oral carbamazepine for about 9 months before the current presentation. One month after initiation, a generalized morbilliform eruption developed, and carbamazepine was stopped. Despite drug discontinuation, the eruption recurred repeatedly over the ensuing 8 months, accompanied by fever (peak 40 °C), cough with white sputum, and arthralgia. Multiple hospitalizations resulted in only transient improvement with moderate-to-high doses of corticosteroids (methylprednisolone sodium succinate up to 40 mg/day); relapse consistently occurred when oral prednisone was tapered below 30 mg/day. Eight days prior to the current admission, the rash flared again with fever (39–42 °C). Parenteral methylprednisolone at an outside hospital controlled the fever but failed to clear the cutaneous lesions, necessitating emergency transfer to our department.

On admission, examination revealed pharyngeal erythema and edema, a diffuse morbilliform eruption involving 60% BSA, bilateral periorbital erythema, facial scaling, bilateral nasal erosions, and inguinal lymphadenopathy. Peripheral blood eosinophils were suppressed to <0.001 × 10^9^/L, which may be explained by prior high-dose corticosteroid exposure. Other laboratory tests indicated impaired hepatic and renal function, as well as an infectious status (**Table 1**). Serology revealed significantly elevated IgG antibody titers of cytomegalovirus, Toxoplasma gondii, herpes simplex virus (HSV) type 1, and rubella virus (yet active reactivation could not be confirmed). HLA-B*15:02 genotyping was also negative. The RegiSCAR score was 6 (definite DRESS), and carbamazepine was determined to be the causative agent.

A multidisciplinary team, including cardiology, nephrology, gastroenterology, pulmonology, and endocrinology, was convened. Methylprednisolone sodium succinate (up to 60 mg/day) was administered with a stepwise taper. A single dose of the etanercept biosimilar 50 mg subcutaneously was given during hospitalization, in addition to ceftriaxone for concurrent infection, hepatoprotective therapy, probiotic supplementation, and topical corticosteroids and antibiotics. Defervescence and progressive resolution of cutaneous lesions occurred during the above treatment. At discharge (hospital day 17), ALT and AST had returned to normal. The patient was discharged with a maintenance regimen of the TNF-α blockade (25 mg subcutaneously twice weekly) and prednisone 20 mg/day. Medications were successfully tapered and discontinued at 3 months post-discharge, with no observed relapse.

#### 3.1.3 Case 3

A 67-year-old woman with a history of trigeminal neuralgia began oral carbamazepine approximately 3 weeks before the index presentation. Approximately 2 weeks into treatment, scattered erythema with pruritus developed and rapidly disseminated over several days, accompanied by fever, chills, malaise, arthralgia, facial edema, cough with sputum, and gastrointestinal symptoms. Five days prior to admission, a dexamethasone local block injection at a regional hospital paradoxically worsened the condition. Three days before admission, an emergency evaluation at our hospital resulted in treatment with intravenous methylprednisolone sodium succinate (40 mg/day × 2 days) and piperacillin-tazobactam. While facial edema and pruritus partially improved, cutaneous lesions persisted, prompting inpatient dermatology admission.

Physical examination revealed a diffuse eruption of erythema involving about 55% BSA with poorly defined borders, locally elevated skin temperature over the inflamed areas, and perioral erosions (**Figure 1**). No significant epidermal necrolysis or severe mucosal ulceration was observed. Laboratory tests revealed marked leukocytosis with eosinophilia, hepatic dysfunction, elevated LDH and γ-GT, severely elevated CD4^+^ and CD8^+^ T cells, and hyper-IgE (**Table 1**). Additionally, HLA-B*15:02 genotyping was also negative. The RegiSCAR score was 5 (probable DRESS), and carbamazepine was identified as the causative agent.

Carbamazepine was discontinued immediately. Treatment comprised intravenous methylprednisolone sodium succinate (maximum 60 mg/day) with a gradual taper, the etanercept biosimilar 25 mg subcutaneously twice weekly for one week, piperacillin-tazobactam for concurrent skin and pulmonary infection, hepatoprotective therapy, proton pump inhibition, antihistamines, and topical corticosteroids. Cutaneous lesions progressively regressed after commencement of the combined regimen, facial edema and pruritus substantially improved, and hepatic enzymes declined steadily. Maintenance therapy with etanercept biosimilar (25 mg twice for one week) was initiated, and prednisone 40 mg daily was prescribed with planned tapering. Follow-up at 2 months confirmed no residual cutaneous lesions.

### 3.2 Literature review

A review of previously published cases identified 8 cases in which TNF-α inhibitors were used for the treatment of DRESS (**Supplementary Figure 1**). The implicated drugs were heterogeneous and included lithium carbonate, co-trimoxazole, vemurafenib, corticosteroids, allopurinol, dabrafenib/trametinib, etc (**Table 2**). RegiSCAR scores, when reported, generally supported probable or definite DRESS.

**Table 2:** Summary of published cases of DRESS syndrome treated with TNF-α inhibitors.

Author (year)	Sex/Age	Causative drug	RegiSCAR score^a^	Organ involvement	Failed prior therapy	TNF-α inhibitor	TNF-α inhibitor regimen	Other therapy	Outcome	Adverse events
Leman et al. (2017) (17)	F/31	Lithium carbonate	6 (Definite)	Liver	None	Recombinant human TNF receptor-IgG fusion protein	50 mg first, then 25 mg every 3 days (total 5 injections)	NR	Remission	NR
Chua et al. (2018) (9)	F/14	Co-trimoxazole	NA	Liver	MP	Infliximab	NA	MP, prednisolone, azathioprine, cyclosporine, mycophenolate mofetil	No clinical response	NR
Maximova et al. (2020) (18)	F/41	Vemurafenib	7 (Definite)	Liver, lung, kidney	Prednisone	Infliximab	5 mg/kg, single dose	Tocilizumab, cortisone	Remission	NR
Kim et al. (2022) (19)	F/26	Corticosteroids	NA	Liver, heart	MP	Etanercept	50 mg weekly ×4	NA	Remission	NR
Vílchez-Sánchez et al. (2022) (10)	F/51	Allopurinol	5(Probable)	Liver	Prednisone, MP	Etanercept	50 mg first, 25 mg 3 days later	Prednisone	Remission	NR
Yordanova et al. (2023) (20)	F/59	Dabrafenib, trametinib	NA	Liver, lung, kidney	MP, IVIG	Etanercept	50 mg single dose	None	Remission	NR
Shen et al. (2023) (12)	F/38	Propylthiouracil	NA	Liver, lung, heart	None	Adalimumab	80 mg first, then 40 mg every 3 days	MP, plasmapheresis	Remission	Symptoms worsen after the first injection of adalimumab
Zhang et al. (2025) (6)	F/61	Mexiletine	4(Probable)	Liver, lung, kidney	MP	Recombinant human TNF receptor-IgG fusion protein	50 mg first, then 25 mg every 3 days	MP	Remission	NR

F, female; IVIG, intravenous immunoglobulin; MP, methylprednisolone sodium succinate; NA, not available; NR, not reported; RegiSCAR, European Registry of Severe Cutaneous Adverse Reactions.

^a^ Reported by the original authors.

All published patients had visceral involvement, most commonly hepatic injury, with variable involvement of the lung, kidney, or heart. The most commonly used TNF-α inhibitor was etanercept (3/8, 37.5%), followed by recombinant human TNF receptor-IgG fusion protein (2/8, 25.0%), infliximab (2/8, 25.0%), and adalimumab (1/8, 12.5%). TNF-α blockade was usually administered after inadequate response to systemic corticosteroids and/or other immunomodulatory therapies, although a few cases (2/8, 25.0%) received TNF-α inhibition earlier in the disease course (**Table 2**).

Overall, clinical improvement or remission was reported in most cases (7/8, 87.5%), whereas one pediatric infliximab-treated case showed no apparent clinical response (9). Additionally, with the exception of a single patient whose symptoms aggravated following the initial adalimumab dose (12), no treatment-related adverse reactions were noted in any other patient.

## 4 Discussion

This case series describes the use of an etanercept biosimilar (recombinant human TNFR2-Fc fusion protein) as adjunctive therapy in three Chinese patients with carbamazepine-induced DRESS. Three patients shared inadequate responses to corticosteroid-based therapy, and the addition of TNF-α blockade was temporally associated with clinical stabilization. To our knowledge, this is the first case series specifically documenting the use of a TNF-α inhibitor for carbamazepine-induced DRESS in a Chinese cohort, and it adds incremental real-world experience to the limited body of literature on TNF-α blockade in DRESS.

The therapeutic rationale for TNF-α blockade in DRESS is anchored in the central role of TNF-α within the cytokine cascade that drives this condition. DRESS is now understood as a multi-organ antiviral T-cell response in which drug-specific CD4^+^ and CD8^+^ T cells, in the potential context of latent HSV reactivation, generate a polyclonal cytokine storm dominated by TNF-α, IFN-γ, IL-5, IL-6, and the type-2 cytokines IL-4 and IL-13 (4, 13, 14). TNF-α produced by activated T cells, macrophages and keratinocytes drives keratinocyte apoptosis, amplifies endothelial activation, and sustains visceral inflammation through induction of NF-κB signaling and recruitment of eosinophils (4, 13). Although reports of TNF-α inhibitor use in DRESS remain scarce, agents such as etanercept have been shown to improve outcomes in other SCARs, including Stevens-Johnson syndrome/toxic epidermal necrolysis (SJS/TEN), by reducing TNF-α and granulysin levels in blister fluid and plasma, expanding regulatory T-cell populations, and thereby decreasing mortality (15, 16). The shared dependence on T-cell-driven proinflammatory cytokine signaling provides a mechanistic bridge to DRESS, and supports the biological plausibility of etanercept as a corticosteroid-sparing adjunct in this setting.

Previous reports of TNF-α inhibitors in DRESS have remained limited to scattered case reports involving heterogeneous causative drugs (6, 9, 10, 12, 17–20). Kim et al. reported successful treatment of a corticosteroid-induced DRESS patient with four weekly subcutaneous doses of etanercept 50 mg, with stabilization of vital signs within 3 days (19). And Chua et al. reported the first pediatric use of infliximab for refractory DRESS, although no therapeutic effect was observed (9). In the vast majority of reported cases, TNF-α inhibitors demonstrated efficacy in DRESS without associated adverse events. Our series complements these reports by focusing exclusively on carbamazepine, the prototypical Asian-population DRESS trigger, and by including the corticosteroid-dependent relapsing phenotype in which TNF-α blockade enabled durable disease control and a planned corticosteroid taper. The use of an accessible biosimilar etanercept formulation also contributes practical, real-world evidence relevant to settings where biologic access has historically been constrained.

Each of our three patients illustrates a distinct potential niche for TNF-α blockade. In Case 1, fulminant DRESS with peak ALT exceeding 30-fold the upper limit of normal and progressive multi-organ involvement that had not been controlled by high-dose methylprednisolone and IVIG, the addition of TNF-α blockade was followed by progressive normalization of hepatic enzymes and clearance of cutaneous lesions. In Case 2, an 8-month corticosteroid-dependent relapsing course, the TNF-α inhibitor allowed durable disease control while permitting safe corticosteroid tapering. In Case 3, a woman with markedly elevated total IgE (1, 103 IU/mL) and substantial T-cell expansion, a brief one-week course of etanercept produced rapid clinical resolution without recurrence at one-month follow-up. DRESS syndrome exhibits considerable clinical heterogeneity, and personalized treatment strategies tailored to distinct phenotypes require further investigation. Fever in DRESS reflects the underlying hypersensitivity reaction rather than an independent process. In our cases, defervescence was achieved through culprit drug withdrawal and systemic therapy. To avoid drug-induced sensitization or exacerbation of DRESS, the introduction of new medications should minimized (21). Antipyretics were withheld, while physical cooling and adequate hydration were provided as supportive care.

Several limitations should be acknowledged. First, the sample size is small and uncontrolled, precluding causal attribution of clinical improvement to TNF-α inhibitor rather than to concomitant corticosteroids, drug withdrawal, or natural disease evolution. Second, reactivation of HHV-6 and HHV-7 is also implicated in the pathogenesis of DRESS and carries prognostic significance (4, 22, 23), which was not systematically assessed in our cohort. Third, as TNF-α inhibition is the mechanistic basis of the proposed intervention, the absence of serial serum TNF-α and broader cytokine measurements is a substantial limitation, which weakens the mechanistic interpretation of the clinical observations. Future studies should incorporate serial cytokine profiling to link clinical response to TNF-α neutralization. Additionally, more precise dosing regimens and treatment protocols remain to be explored in future studies. While the diagnosis was supported by RegiSCAR scoring (5–9) and a coherent clinical-laboratory pattern, a biopsy might have provided additional histopathologic validation. Finally, regarding the literature review, inconsistent reporting standards across prior cases limited the availability of patient information and precluded further in-depth analysis of these cases.

## 5 Conclusion

This study reviewed previously published cases alongside our single-center data to explore the potential effectiveness and safety of TNF-α inhibitors in DRESS syndrome. In our case series, adjunctive TNF-α blockade with an etanercept biosimilar was temporally associated with rapid clinical stabilization, progressive resolution of cutaneous lesions, and improvement of hepatic injury in three Chinese patients with carbamazepine-induced DRESS who had shown inadequate or transient responses to corticosteroid-based therapy. Despite a small sample size and concomitant treatments, these findings suggest that TNF-α inhibition may offer potential benefit and an acceptable short-term safety profile in this limited cohort of selected patients with severe, refractory, or corticosteroid-dependent DRESS. Further prospective studies are required to define optimal patient selection, timing, dosing, and safety monitoring.

## Data Availability

The original contributions presented in the study are included in the article/**Supplementary Material**. Further inquiries can be directed to the corresponding authors.
