# Supplementary material for: TNF-α blockade for carbamazepine-induced drug reaction with eosinophilia and systemic symptoms
Source: Front Immunol. 2026 Aug 3;17:1872304. doi: 10.3389/fimmu.2026.1872304 (PMC13477922; doi:10.3389/fimmu.2026.1872304)
Supplement: Supplementary file 1 [file Table1.docx]

**Supplementary Table 1.** Full electronic search strategy (databases searched from inception to 1 July 2026).

| Database | Search string |
| --- | --- |
| PubMed | ("drug reaction with eosinophilia and systemic symptoms"[tiab] OR "DRESS"[tiab] OR "drug-induced hypersensitivity syndrome"[tiab] OR "DIHS"[tiab] OR "drug hypersensitivity syndrome"[tiab]) AND ("tumor necrosis factor"[tiab] OR "tumour necrosis factor"[tiab] OR "TNF"[tiab] OR "TNF-alpha"[tiab] OR "TNF-α"[tiab] OR etanercept[tiab] OR infliximab[tiab] OR adalimumab[tiab] OR golimumab[tiab] OR certolizumab[tiab] OR "TNF inhibitor*"[tiab] OR "TNF antagonist*"[tiab] OR "TNF blocker*"[tiab]) |
| Web of Science | TS=(("drug reaction with eosinophilia and systemic symptoms" OR DRESS OR "drug-induced hypersensitivity syndrome" OR DIHS OR "drug hypersensitivity syndrome") AND (etanercept OR infliximab OR adalimumab OR golimumab OR certolizumab OR "anti-TNF" OR "anti TNF" OR "TNF inhibitor*" OR "TNF antagonist*" OR "TNF blockade" OR "tumor necrosis factor inhibitor*" OR "tumour necrosis factor inhibitor*")) |

Reference lists of all eligible articles were additionally hand-searched. Two reviewers (M.Z. and K.Z.) independently applied the eligibility criteria, and disagreements were resolved by consensus with a third reviewer (W.L.).

**Supplementary Table 2.** Individual RegiSCAR component scores for the three institutional cases

| RegiSCAR component | Case 1 | Case 2 | Case 3 |
| --- | --- | --- | --- |
| Fever ≥38.5°C (yes 0 / no –1) | 0 | 0 | –1 |
| Lymphadenopathy (yes 1 / no 0) | 1 | 1 | 0 |
| Eosinophilia (≥1.5×10⁹/L or ≥20%: 2; 0.7–1.499×10⁹/L or 10–19.9%: 1) | 2 | 0 | 2 |
| Atypical lymphocytes (yes 1 / no 0) | 1 | 0 | 0 |
| Skin extent >50% BSA (yes 1 / no 0) | 1 | 1 | 1 |
| Skin rash suggestive of DRESS (≥2 features: 1) | 1 | 1 | 1 |
| Biopsy suggesting DRESS (yes/unknown: 0; no –1) | 0 | 0 | 0 |
| Organ involvement (1 organ: 1; ≥2 organs: 2) | 2 | 2 | 1 |
| Resolution ≥15 days (yes 0 / no –1) | 0 | 0 | 0 |
| Other causes excluded (antinuclear antibody, blood culture, serology for HAV/HBV/HCV, chlamydia/mycoplasma, none positive and ≥ 3 of above negative: 1) | 1 | 1 | 1 |
| Total score | 9 (definite) | 6 (definite) | 5 (probable) |

BSA, body surface area; RegiSCAR, European Registry of Severe Cutaneous Adverse Reactions.

Final score 2–3, possible case; final score 4–5, probable case; final score > 5, definite case.

**Supplementary Figure 1.** Flowchart of study selection

**Identification of studies via databases**

Records identified from databases (n = 156)

PubMed (n=65)

Web of Science (n=91)

Duplicate records removed (n = 18)

**Identification**

Records screened

(n =138)

Records excluded by reviewing title and abstract

(n = 97)

Reports sought for retrieval

(n =41)

Reports not retrieved

(n = 2)

**Screening**

Reports assessed for eligibility

(n = 39)

Reports excluded (n= 31)

- Not meeting diagnostic criteria (n = 12)
- Not related to the treatment with TNF - α blockers (n = 19)

Studies included in review

(n = 8)

**Included**
